# Small interfering RNA: From designing to therapeutic in cancer

**DOI:** 10.1016/j.jgeb.2025.100484

**Published:** 2025-04-03

**Authors:** Jyoti Singh, Abdulaziz S. Saeedan, Gaurav Kaithwas, Mohd Nazam Ansari

**Affiliations:** a Department of Pharmaceutical Sciences, School of Pharmaceutical Sciences, Babasaheb Bhimrao Ambedkar University (A Central University), Vidya Vihar, Raebareli Road, Lucknow 226025 Uttar Pradesh, India; b Department of Pharmacology and Toxicology, College of Pharmacy, Prince Sattam Bin Abdulaziz University, Alkharj 11942, Saudi Arabia

**Keywords:** Cancer, Hypoxia, RNAi, siRNA, siRNA modification, Clinical trials

## Abstract

Cancer has become a significant public health concern worldwide. It is a group of diseases, often resulting from the dysregulation of multiple cellular pathways involved in differentiation, cell proliferation, cell cycle regulation, and DNA repair. These disruptions are primarily caused by genetic mutation and epigenetic alterations which lead to uncontrolled growth and tumor formation. Targeted therapy is a precise and effective strategy to overcome the shortcomings of conventional therapy. RNA interference (RNAi) is a gene-silencing mechanism that has an uncanny ability to target disease-associated genes. Small interfering RNA (siRNA) is a key component of RNAi and has shown promise in silencing oncogenes and inhibiting cancer progression. However, the therapeutic application of siRNA faces several challenges such as poor cellular uptake, short half-life, endosomal escape, immune system activation, and off-target. Strategies to address these challenges are optimized designing of siRNA, advanced delivery systems, and chemical modification to improve cellular uptake and protect from degradation. This review focuses on the therapeutic potential of siRNA in cancer treatment and discusses the action mechanism of siRNA, barriers in siRNA, and strategies to overcome them. The review shed light on the current clinical trial of siRNA-based cancer therapy, along with outcomes and limitations.

## 1 Introduction

Cancer is a complex and heterogeneous group of diseases resulting from the dysregulation of multiple cellular pathways, including differentiation, proliferation, cell cycle regulation, and DNA repair. These disruptions, driven by genetic mutation, epigenetic modifications, and environmental factors, lead to uncontrolled growth. This enables cells to escape from homeostatic control that normally suppresses uncontrolled cell proliferation and prevents aberrant cell survival outside their normal niche. This leads to the formation of masses of cells called tumors, which can be further subdivided as either benign or malignant in nature^1^. A benign tumor remains localized, whereas malignant tumors metastasize to distant organs and infiltrate surrounding tissues, organs, and blood vessels.^2^ The disordered and abnormal mass of cells results in an oxygen (O_2_) gradient inside the microenvironment^3^. The inadequate blood supply to swiftly multiplying cancer cells results in oxygen deprivation. Cancer cells utilize diverse metabolic mechanisms to circumvent oxygen shortage^2^. Inadequate oxygen levels at the cancer site create a hypoxic environment. Hypoxia is a fundamental characteristic associated with all forms of cancer. This condition usually occurs in case of a 1 mm increased tumor size. It is generally accepted that a hypoxic tumor is associated with poorer outcomes^4^.

Despite advancements in conventional cancer therapies such as chemotherapy, radiation, and immunotherapy, these treatments often have limitations. There is a requirement for new strategies that enhance effectiveness and reduce challenges. One approach is RNAi, a gene silencing mechanism, to target oncogenes and tumor-associated biomarkers. In recent years siRNA has gained considerable attention in clinical utilization. For example, Patisiran, a siRNA-based gene therapy, treats amyloidotic polyneuropathy, by silencing Transthyretin (TTR) gene^5^, and Givosiran is also a siRNA-based gene drug to cure acute hepatic porphyria by targeting δ-aminolevulinate synthase 1 (ALAS1)^6^. In cancer research, treatments like siG12D-LODER, designed to target the KRAS mutation in pancreatic cells, and TKM-PLK, developed to silence polo-like kinase1 in solid tumors have shown promise in clinical trials[7], [8]. However, RNAi has faced prominent drawbacks such as short half-life due to nucleases degradation and clearance by reticuloendothelial cells, endosomal escape, off-targets leading to mutation in normal cells, low cellular uptake due to the negative charge^9^]. To overcome these limitations advanced delivery systems, including lipid nanoparticles, polymeric carriers have been explored with potential benefits^10^]. Further siRNA therapy showed improved therapeutic efficacy and reduced resistance when used in combination with chemotherapy and immunotherapy^11^. For instance, a preclinical study demonstrated that blocking STAT3 using locally delivered CpG-Stat 3 siRNA significantly improved the efficacy of immune checkpoint inhibitors in B cell lymphoma and melanoma models^12^. Another study demonstrated that Survivin siRNA with neoadjuvant chemotherapy (paclitaxel or epirubicin) showed significant apoptosis and improved drug sensitivity in MCF-7 breast cancer cells^13^. The coming sections of the review include detailing the process of RNAi, its componentizing, mechanism, shortcomings of siRNA therapeutics, siRNA design, strategies for enhancing stability, and delivery, and address the challenges in achieving effective gene silencing. The review aims to provide insights into recent clinical studies while discussing the potential therapeutic applications and limitations of siRNA-based treatments in cancer.

## 2 Mechanisms of cancer progression

**Fig. 1:**
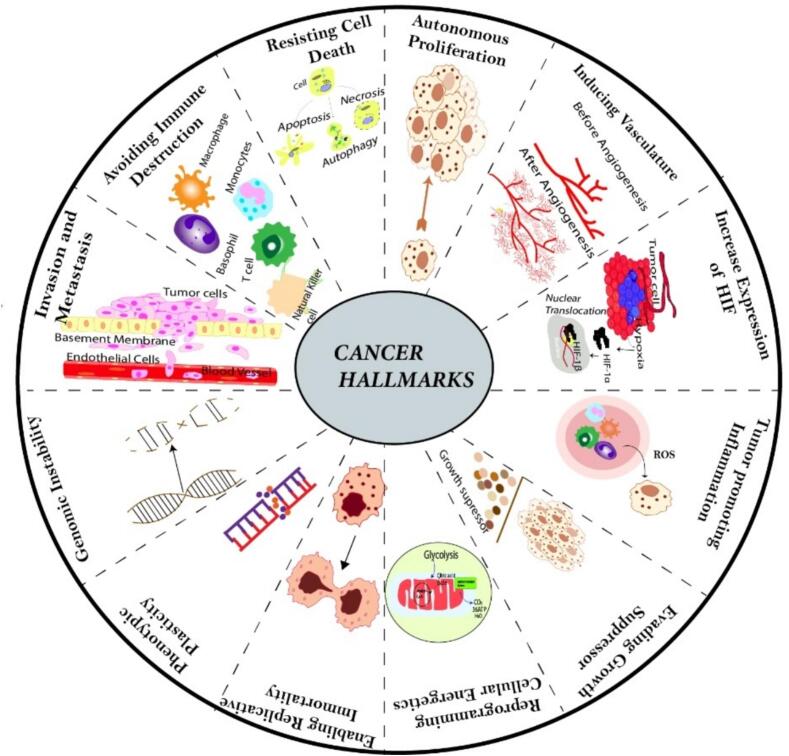
Hallmarks of cancer represents the different hallmarks like avoid cell death, sustaining proliferative signal, angiogenesis, genomic instability, activation of invasion of metastasis etc. possessed by most cancers.

Hanahan and Weinberg identified six key functional features of cancer. These characteristics include the ability to generate their own growth signals, resistance to anti-growth signals, evasion of apoptosis, continuous angiogenesis, metastasis, and uncontrolled capacity for replication Fig. 1.

**Fig. 2:**
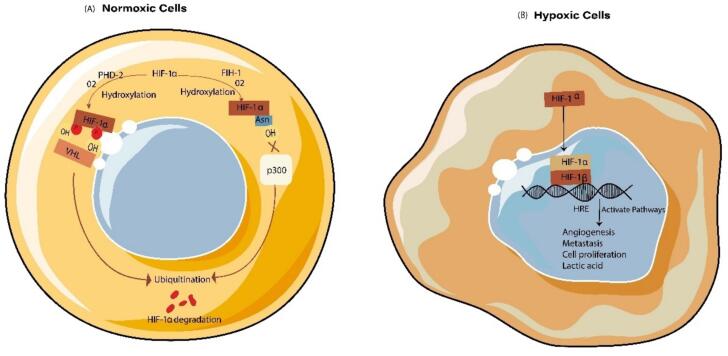
A graphical diagram represents the pathway of HIF-α activation in hypoxia. a). In normoxic cells, in the presence of O_2_, activation of PHD and FIH (O_2_-dependent enzymes) causes the hydroxylation of proline (P) and aspargine (Asn) residues. Hydroxylation of these residues causes their ubiquitylation by pVHL and results in the proteasomal degradation of HIF-α b). In hypoxic cells, HIF-α gets dimerized with HIF-β and the residue Asn interacts with the p300 transcriptional cofactor.

One of the major features of solid tumors supporting the above six key functional characteristics is hypoxia. Hypoxia in cancer is classified into chronic hypoxia and cyclic hypoxia. Chronic hypoxia occurs in early tumor development when blood vessels supply oxygen insufficient oxygen. Hypoxia induces the activation of transcriptional factor, hypoxia-inducible factor (HIF). HIF is a heterodimer composed of HIF-α and HIF-β subunits. HIF-α is regulated by oxygen and expressed in hypoxic conditions, but the HIF-β subunit is constitutively expressed and unaffected by oxygen levels^14^. In normoxic cells, HIF-1α is degraded by enzymes prolyl hydroxylase (PHD)^15^and factor inhibiting hypoxia-inducible factor (FIH) (Fig. 2a)^16^. PHD facilitates von Hippel-Lindau protein (pVHL) mediated ubiquitylation, resulting in proteasomal degradation of HIF-1α. At the same time, FIH hydrolyzes Asn803 of HIF-1α and Asn847 of HIF-2α to prevent the interaction between p300 and HIF-α[17], [18]].

In hypoxic cells, PHD and FIH become inactivated, resulting in elevated HIF-α accumulation. HIF-1 α accumulates maximally during the initial 4 h of chronic hypoxia, following which its levels decline rapidly (Fig. 2b) and induce the transcription of several genes to modulate metabolism, metastasis, angiogenesis, lymphangiogenesis, immune evasion, and functions of chemokines to nourish the fast-growing tumor^19^. Angiogenesis (development of new blood vessels) is a prerequisite for survival and the development of tumor cells. HIF-1α upregulates the expression of several chemokines[19], [20] which in turn upregulates the expression of vascular endothelial growth factor (VEGF) to promote angiogenesis at the site of the tumor. The consequences of HIF-1α activation are discussed in the. While hypoxia is the universal hallmark of cancer other factors such as genetic instability and evasion of apoptosis also play an important role in cancer progression. Tumor cells appear more genetically unstable compared to the normal cells. Genetic instability includes small structural variations like increased frequency of base pair mutation, microsatellite instability, and changes in chromosome number and structure. Mutation in tumor suppression genes like TP53 plays a critical role in driving unchecked cell division. The p53, which encodes the TP53 gene, is involved in a major defense barrier in cancer. Often referred to as the guardian of the genome, p53 is the most frequently mutated gene in cancer. P53 regulates genomic instability by regulating the cell cycle, DNA repair, and cell cycle^21^. P53 is also involved in epigenetic alteration where it modulates DNA methylation and histone modifications. For instance, P53 regulates one-carbon metabolism by controlling Slc43a2, a methionine transporter, which influences histone methylation. Its depletion mimics p53 loss which leads to chromosomal abnormalities and altered transcriptional control of repetitive regions^22^. Cancer cells evade apoptosis by disturbing both extrinsic and intrinsic apoptotic pathways. In the extrinsic pathway, downregulation of Fas/TNF receptors occurs which further blocks caspase 8 activation. In the intrinsic pathway upregulation of anti-apoptotic proteins (BCL-2, BCL-XL, MCL-1) and downregulation of pro-apoptotic proteins (BAX, BAK, APAF-1) to block the cytochrome *C* release. Moreover, increased expression of IAPs such as XIAP suppresses caspase 3,7, and 9 which further inhibit apoptosis. These adaptations promote tumor survival, metastasis, and therapy resistance^23^].

### 2.1 RNA interference

RNAi is a highly conserved post-transcriptional gene silencing process that selectively degrades target mRNA thereby suppressing the gene expression. The whole mechanism happens in the cytoplasm and is initiated when siRNA is incorporated in RNA-induced silencing complex RISC. The antisense strand of siRNA guides RISC to the complacentry sequences of the mRNA target, where Argonaute 2(Ago2), the catalytic component of RISC, cleaves the mRNA, leading to mRNA degradation and preventing protein synthesis. This sequence-specific degradation makes siRNA a powerful genetic tool for therapeutic application mainly in cancer treatment.

**Fig. 3:**
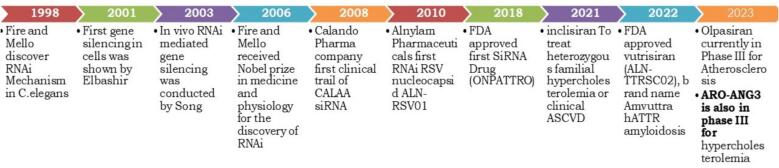
Timeline of RNAi discovery.

RNAi is a biologically conserved process observed in all eukaryotic creatures, encompassing insects, fungi, plants, and animals. Primarily, non-coding RNAs of around 20–40 nucleotides facilitate gene silencing in a highly sequence-specific manner^24^. In 1990, Jorgensen and co-workers first reported this phenomenon in plants^25^. Afterward, in 1998, Fire and co-workers revealed the gene silencing potential of ds-RNAs in nematode *Caenorhabditis elegans*^24^*]*. It is a naturally occurring phenomenon but, in 2001, Elbashir et al. studied the gene silencing effect of 21 nucleotides long synthetic siRNA in mammalian cell lines; HeLa, and human HEK^26^). Hence, RNAi discovery in mammalian cells attracted great interest to explore its therapeutic potential against diabetes, cardiovascular abnormalities, and viral diseases such as viral hepatitis[27], [28], [29]. To date FDA has approved six siRNA therapeutics: Patisiran, Givosiran, Lumasiran, Inclisiran, Vutrisiran, and Nedosiran with many more in clinical trials Fig. 3^30^. Preclinical studies have demonstrated siRNA potential in silencing genes related to angiogenesis, immune evasion, proliferation. For example, siRNA targeting mutant KRAS is effective in inhibiting tumor cell proliferation without affecting normal cells. The inhibition of the PARP gene can make cancer cells more susceptible to DNA-damaging agents while siRNA against BCL2 enhances chemotherapy-induced apoptosis^31^. Moreover, siRNA against VEGF showed better outcomes when conjugated with PEI modified with a fluorine-containing alkyl chain that brings hydrophobic and oleophobic characteristics to its surface[32], [33]. However, siRNA-based therapy has its challenges to translate as an entirely acceptable gene therapeutic. Nonetheless, siRNA-based therapy faces challenges in being fully accepted as a gene therapeutic. Challenges include the effective delivery of siRNA into the cytoplasm, where it interacts with RISC to form a complex, a process primarily hindered by endosomal entrapment. Additional challenges include off-target effects, where siRNA binds to unintended mRNA sequences; immunogenic responses is another one, where siRNA is recognized as a foreign agent by the immune system; degradation by nucleases; and high instability^34^. To address these challenges, various delivery systems and chemical modifications have been introduced. These barriers and challenges are discussed in detail in different sections.

### 2.2 Core components of RNAi

The Dicer protein is the ribonuclease III enzyme and initiating element of the RNAi mechanism by processing long double-strand RNA and hairpin RNA precursors into small regulatory RNAs (siRNA, miRNA). It is fundamentally a multi-domain protein composed of an N-terminal RNA helicase, two RNase III domains, a double-stranded RNA binding domain (dsRBD), and PAZ domains^35^. Another crucial component of the system is the Ago protein family, a highly specialized module for binding short RNAs. Notably, of the four subfamilies of Ago proteins (Ago1, Ago2, Ago3, Ago4), only Ago2 possesses the endonuclease activity to cleave target mRNA^36^. The four Ago proteins are structurally very similar, however, they contain nonconserved amino acids. Ago1 is involved in miRNA-mediated gene silencing and heterochromatin formation, Ago3 contributes to neurodevelopment and small RNA binding, and Ago4 specializes in RNA-directed DNA methylation and antiviral defense^37^. Similar to Dicer, Ago is a multi-domain protein with N-terminal, PAZ, MID, and PIWI domains^35^. Across all domains, the N-terminal domain is crucial for the unwinding of double-stranded siRNA, whereas the PIWI domain is vital for the cleavage of target mRNA due to its endonuclease enzymatic activity[,[36], [38], [39]. The PAZ and MID domains are binding domains, where the PAZ domain anchors the 3́–OH end of siRNA, and the MID domain binds with the 5′ phosphate end^40^].

### 2.3 Small non-coding RNAs

**Fig. 4:**
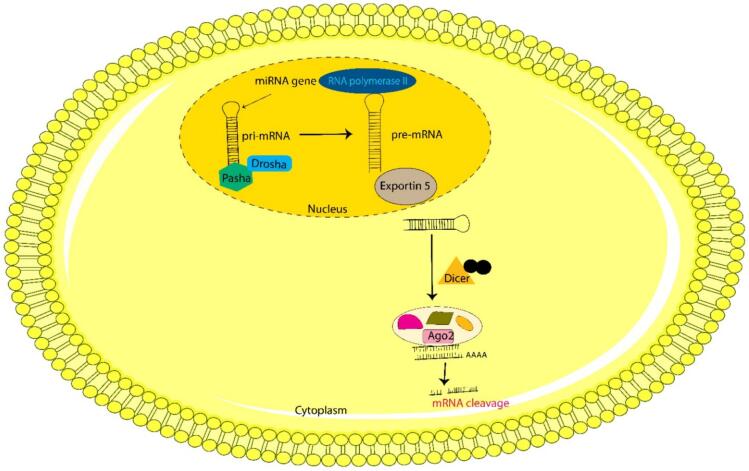
An Illustration of the biogenesis of miRNA. Pre-miRNA is generated in the nucleus from pri-miRNA and then exported in the cytoplasm, where dicer dices it into mature miRNA.

RNAi is mediated by small non-coding RNAs. These are small ds-RNAs with varying lengths of ∼ 20–40 nucleotides. There are two major classes of small regulatory RNAs *viz;* siRNA and miRNA. Endogenously expressed, single-stranded miRNAs perform a crucial role in the regulation of cell differentiation, proliferation, and their survival; thereby regulating mRNA degradation or translational inhibition^41^]. miRNAs are believed to govern the gene expression involved in numerous physiological and developmental pathways^42^. Inside the nucleus, miRNA genes are acted upon by RNA polymerase II enzyme and in turn transcribed into several hundred nucleotides-long primary miRNAs (pri-miR)^43^. These pri-miR transcripts contain one or more hairpins, while the mature miRNAs are in their double-stranded stem. Afterward, the mature miRNA is generated from pri-miRNAs by successive action of RNase III-like enzymes Drosha and Pasha inside the nucleus, and Dicer in the cytoplasm. The nuclear microprocessors Drosha and Pasha process pri-miR into 70 nucleotide-long stem-loop pre-miRNA. Now, this pre-miRNA is exported into the cytoplasm with the help of RanGTP-dependent nucleo-cytoplasmic cargo transporter, Exportin 5. Further, dicer dices this pre-miRNA into ∼ 22 nucleotide long mature miRNA duplexes[42], [43]. One strand which incorporates onto the Ago protein is known as guide miRNA, while the other is cleaved by PIWI domain of Ago protein and is known as passenger strand^44^]. The biogenesis of miRNA is explained in Fig. 4.

In mammals, siRNAs are exogenously introduced in the cytoplasm. Some structural and biochemical studies have reported that both siRNAs and miRNAs share similar protein machinery and gene silencing pathways[45], [46]. Therefore, the extent of complementarity between miRNA/siRNA and their target mRNAs dictates the mechanism of gene silencing[47], [48]. siRNA which binds perfectly to its target, directs m-RNA cleavage via RISC. However, miRNA which binds partially to its target leads to translational repression instead of cleavage.[47], [49]]. A single miRNA can regulate multiple m-RNA sequences due to the imperfect base pairing^50^]. Gene silencing mechanism through miRNA is explained along with siRNA gene silencing mechanism.

### 2.4 siRNA-mediated gene silencing

As briefed above, siRNA is a non-coding regulatory ds-RNA of ∼ 20–23 nucleotide base pairs in length with 2-nt overhangs on 3́-ends. As per its name, siRNA interferes with the gene expression of a gene with a full complementary sequence. The proposed model for siRNA-mediated gene silencing is explained in Fig. 4. Dicer processes the long double-stranded RNA, hairpin miRNA into siRNA which in turn incorporates onto RISC (RNA-induced silencing complex) and activates the RISC machinery to inhibit the gene expression. Therefore, RISC assembly is crucial to trigger RNAi. RISC assembly is a highly systematic pathway. In this pathway, siRNA duplexes are structurally altered till the formation of mature, active, and functional RISC. RISC assembly occurs in two steps; **(i) RISC loading:** In this stage, the short RNA duplexes are assigned to Ago proteins according to the asymmetry principle, which is determined by the stability of their 5′-ends. The less stable 5́-end is designated as the antisense or guide strand, whereas the strand with the stable 5́-end is referred to as the passenger strand. In addition to thermodynamic stability, the identity of the 5′ end nucleotide (recognized by the MID domain of AGO) also affects strand selection. The TRBP functions as a thermodynamic asymmetry sensor and facilitates the efficient loading of siRNA into RISC. TRBP is a protein with three double-strand RNA binding domains, it is a component of Dicer. Protein kinase RNA (PKR) activator (PACT) plays a crucial role in siRNA stability and RISC loading. TRBP and PACT are critical for efficient miRNA processing because their depletion results in decreased mature miRNA levels and impaired gene silencing. Moreover, chaperone machinery including HSP90 and HSP70 along with their co-factors assist in stabilizing Ago2 in its active conformation. RISC loading complex plays a vital function in the effective transfer of nascent miRNAs or siRNAs from Dicer to Ago thereby enabling the gene silencing process^51^]. **(ii) Strand dissociation:** This is the concluding phase of the RISC assembly. At this stage, siRNA duplexes are dissociated within Ago. Duplex separation can be achieved by two methods. One is Ago 2 dependents cleavage where the PIWI domain of the Ago2 protein triggers the cleavage of either the sense or passenger strand. Another is Ago proteins (Ago1, Ago3, and Ago4) mediated unwinding siRNA duplexes where wedging of the N domain causes it to insert between the siRNA duplexes, resulting in their opening and subsequent unwinding. The Ago complex containing short RNA duplexes is referred to as pre-RISC.

But, after the dissociation of the passenger strand, the complex with a guide strand only is termed as RISC, holo-RISC, or mature RISC^52^. As mature RISC is formed, the antisense strand of siRNA hybridizes with its complementary target mRNA and guides Ago protein for mRNA cleavage. After cleavage of the target mRNA, RISC is recycled and re-entered into the RNAi machinery pathway to carry out a similar event multiple times^47^]. Different siRNAs have their own loading efficiency and hence have differential potential on their gene silencing effect. The α-form helix is supposed to have better and more stable performance in contrast to the β-form helix in RNAi^53^].

### 2.5 Barriers in siRNA therapeutics

#### 2.5.1 Systematic and localized delivery challenge of siRNA

Delivery of siRNA can be broadly categorized into localized and systematic delivery; each has its own challenges and advantages. Systemic delivery faces more barriers compared to localized siRNA delivery. Systemic delivery has various types of delivery challenges which limit its b bioavailability at the target site. Systematic delivery requires targeting internal organs like the spleen and liver for which siRNA must be inside the circulatory system. However intravenous administration of siRNA faced several issue-like nuclease degradation, short half-life, nonspecific binding, renal clearance, immune reaction, etc. The small size, negative charge, and hydrophilic nature of siRNA further hinder its ability to cross cell membrane efficiently Localised delivery of siRNA involves the direct application of therapy to the specific organ or tissues of interest. Organs like eyes, mucus membrane skin, and tumors are more suitable for the localized siRNA. In pulmonary infection, intranasal and intratracheal delivery has shown better response on pulmonary epithelial cells with increased bioavailability which opens the door for the promising treatment of lung disorder. This method provides optimal bioavailability at the intended place and circumvents the obstacles encountered with systemic administration[54], [55]]. In contrast, if naked siRNA is administered intravenously, it is degraded by the endonuclease’s enzymes. The kidney also eliminates the siRNA due to its small size (MW 13 kDa). Due to the negative charge on the siRNA membrane transportation is very difficult. The average half-life of unmodified or naked siRNA is approx. 10–15 min^56^. The first challenge lies in the inherent instability of siRNAs under physiological conditions, making them susceptible to rapid clearance. The physiological environment contains several nuclease enzymes which degrade the siRNA when encountered in the bloodstream.

#### 2.5.2 Clearance by mononuclear system

Systemic delivery of siRNA mainly relies on the carrier system due to the naked siRNA instability. These carriers include liposomes, micelles, nanosphere, nanocapsules, conjugates, microemulsion, and other formulations, which provide a protective covering to siRNA against nuclease degradation and other biological processes. In solid tumors, these carrier-based siRNA therapeutics showed increased permeability and retention effect (EPR)^30^. However, nanosized carriers rapidly distributed to the reticuloendothelial system (RES) and went to the phagocytosis by mononuclear phagocytosis system (MPS). During the phagocytosis clearance process, siRNA interacts with blood components like immunoglobulins of the complement system, leading to its accumulation in RES organs such as the spleen and liver^57^]. High amounts of siRNA cleared by the renal system due to their small size. Unmodified or naked siRNA have a shorter half-life. To enhance siRNA stability and prolong its circulation time surface modification using flexible and hydrophilic polyethylene glycol (PEG) or other surfactants has been employed. These modifications also limit the interaction with proteins thereby protecting from opsinization and immune activation with better carrier stability. PEG also reduces the negative charge on siRNA and enhances siRNA cell uptake. For example, raising PEG2000 concentration with siRNA-lipo complex from 1 to 2 to 5 % completely silenced PETN expression in vitro study by showing prolonged half-life and efficient silencing properties^58^].

### 2.6 Endosomal and lysosomal entrapment

**Fig. 5:**
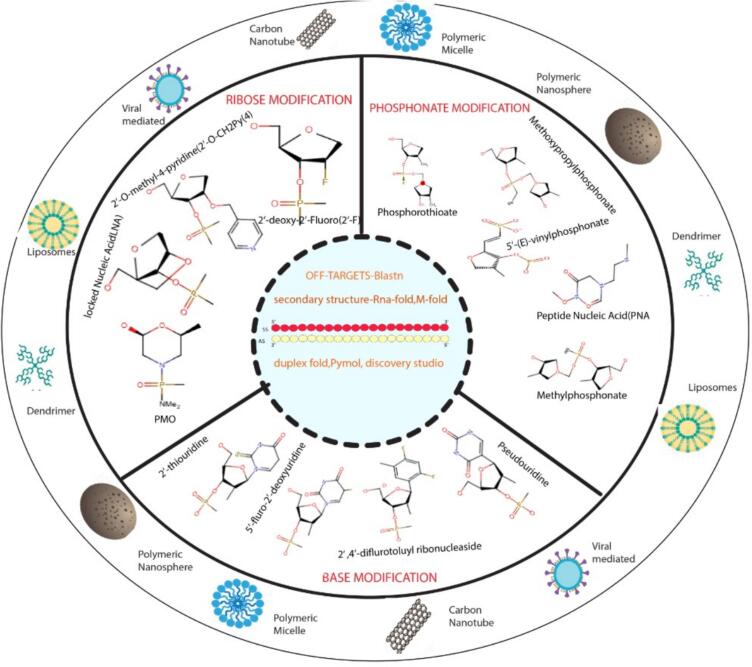
Barriers to siRNA delivery following systemic administration. Extracellular obstacles include enzymatic degradation by nucleases in the bloodstream, rapid renal clearance, and uptake by the reticuloendothelial system (RES). Intracellular challenges involve limited cellular uptake and inefficient endosomal escape, which reduce the effectiveness of siRNA-mediated gene silencing at the target site.

Even if unmodified siRNA successfully reached their target it major challenge remains- endosomal and lysosomal entrapment^59^]. These intracellular organelles contain numerous nucleases and maintain an acidic pH range from 4 to 6. These features make siRNA more susceptible to premature degradation. So there is a requirement for an effective escape strategy from the endosomes and lysosomes so that it can effectively form a complex with RISC^60^. Following cellular uptake siRNA therapeutics went through a series of internal compartments, moving from early endosomes to late endosomes and finally to lysosomes. This transition involves a systematic vesicular maturation. Initially, early endosomes merge with multivesicular endosomes. After that late endosome merges with the lysosome, forming multilamellar hybrid organelles before ultimately becoming a lysosome. The characteristic feature of this maturation process is the steady increase in the acidity within these compartments with pH value continuously decreasing from 7.4 to progressively acidic levels: early endosomes (below pH 6.5), late endosomes (below pH 6.0), and lysosomes (below pH 5.0) Fig. 5^61^]. Many delivery systems have been reported that have pH-sensitive systems through which these systems respond to pH changes by absorbing H + and presenting a positive charge on the system. After that osmatic pressure increases in endosomes and lysosomes due to which Cl − and H2O internally flow out^62^]. This effect is known as the proton sponge effect or colloid osmotic pressure effect. These changes promote the plasma membrane disruption and facilitate the siRNA release to the cytoplasm.

### 2.7 Immunogenicity of siRNA

Toll-like receptors (TLRs), pattern recognition receptors that are expressed on immune cells, are capable of identifying pathogen-associated molecular patterns, such as viral dsRNA and CpG DNA^63^. Nucleotide sequences in siRNAs, including the UG dinucleotide and 5′-UGU-3′ motifs, are specifically recognized by TLR3, TLR7, and TLR8. The innate immune system is activated by siRNAs upon injection into the body, which leads to the production of significant quantities of cytokines. The fourth challenge concentrates on the off-target effects of siRNAs. Even though siRNAs, which are 20 nucleotides in length, exhibit a high degree of specificity in terms of base-complementary pairing, cells contain a significant number of long-stranded mRNAs and miRNAs. The degradation of unintended mRNAs and miRNAs can result from the incomplete complementary pairing of siRNAs, which can contribute to non-specific gene regulatory effects^64^].

### 2.8 Off-target effect

Another significant challenge in siRNA therapeutics is off target effect. Despite siRNA’s high specificity due to base pair complementary, unintended gene silencing can occur due to the partial complementary with non-target mRNA. This phenomenon can lead to nonspecific gene regulation, disrupting cellular function. Leaky vasculature is characteristic of solid tumors, which enhances the EPR effect by providing a pathway for increased drug accumulation in cancer cells compared to normal cells. However, a growing body of evidence showed that long-term EPR effectiveness may be limited, pushing efforts to improve targeted delivery of siRNA^65^]. One promising strategy is exploring surface ligand modifications to enhance receptor-mediated uptake. The goal of this strategy is to improve the delivery of targeted cargo by looking into the overexpression of certain cell receptors that are common in certain types of tumor cells^66^. By functionalizing the carrier surface with ligands that are specific to these receptors, we can achieve a more precise delivery of siRNA without off-target effects. To lessen the off-target effect, different methods are used, such as receptor-targeted delivery, antibody conjugation, and strategic chemical modifications. The example of conjugating siRNA with N-acetylgalactosamine (GalNAc) enables targeted delivery to liver cells by binding to the asialoglycoprotein receptor (ASGPR) on hepatocytes. This strategy has significantly increased RNAi activity in liver tissue with 5-fold greater improvement in RNAi therapeutics application in vivo. For targeting Transthyretin‐mediated amyloidosis (TTR), a rare progressive disease, siRNA has shown a significant reduction of TTR mRNA^67^. Furthermore, the introduction of PS linkage modification to the GalNAc conjugate has shown protection against the exonuclease activity while ensuring targeted delivery.

### 2.9 Strategy to overcome siRNA challenges

#### 2.9.1 Chemical modification

**Fig. 6:**
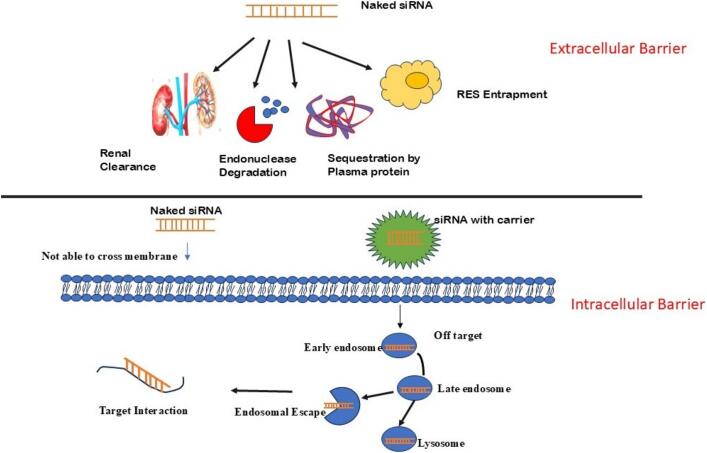
Strategy of siRNA in cancer treatment. The inner circle illustrates the different bioinformatic tools for the optimized design of siRNA to reduce off-target effects. The middle circle showed chemical modifications that involved base modification, phosphate modification, and Ribose modification to increase the stability and half-life of siRNA. The outer circle represents the different delivery vehicles for the target delivery of siRNA. It includes nanoparticles (LPNs, SNPs), lipid-based delivery (cationic lipid, Lipid polyplex), and bi-conjugate (GalNAc conjugates) for the effective treatment of cancer.

Chemical modifications play a pivotal in enhancing the stability and efficacy of unmodified siRNA. These modifications can be implemented directly on siRNA without affecting its ability to silence its target gene. A number of modifications have been introduced across the different segments of siRNA including terminal region, nucleotide, sugar moieties, and nucleotide bases Fig. 6. All these changes help in better cellular uptake and prolong the half-life of designed siRNA. Ribose modification at the 2′-OH position, essential for enzymatic hydrolysis, is the most common and widely used modification to protect siRNA from nuclease degradation. It can be replaced by 2′-O- methyl (2′-O-ME), which is the most frequently used in siRNA modification which significantly increases siRNA stability and half-life^68^. In the same way, a series of analogs have been introduced for ribose modification another example is 2′-O-methoxyethyl showed very good binding affinity to target mRNA and is resistant to nuclease attack. Additionally, 2′deoxy-2′fluoro (2-F’) or 2′-*arabino*-fluoro is also very efficient for both clinical and preclinical applications of siRNA^69^]. Locked nucleic acid (LNA) nucleotides contain a methylene bridge between the 2′ and 4′ carbons of the ribose ring. LNA enhanced the base pairing of guide strands with target mRNA by locking the ribose into its preferred C3′ endo confirmation. It acts as a thermally destabilizing nucleic acid that minimizes the off-target effect by blocking the passenger strand entry and providing conformation support for guide strand loading in the RISC complex. A single LNA enhanced the melting temperature of RNA-RNA duplex up to 5–10 °C^70^. Several phosphate modifications have been introduced to protect siRNA from exonuclease degradation. Phosphorodithioate (PS2), phosphorothioate (PS), methyl phosphonate, and other modifications were introduced for better RNAi activity. The PS linkage is obtained by a sulfur atom replacing one nonbridging oxygen of phosphodiester bond^71^. This modification was initially introduced in antigen oligonucleotide (ASO) and approved by the FDA. It has been observed that siRNAs with PS linkages retain the ability to silence target sequences. However, cytotoxicity was observed when the number of PS linkage was increased throughout the siRNA^72^]. Another example of phosphate modification is boranophosphate linkages obtained by introducing a boron atom in place of nonbridging oxygen atoms of phosphodiester bonds. This adjustment was effective but did not garner extensive scientific validation due to the lack of confirmation for this modification^73^. Incorporating Single –(s)- glycol nucleic acid(S-GNA) nucleotide at the 7 position of the antisense strand of siRNA showed reduced off-target effect in rodent models. Moreover, in clinical trials, S-GNA-modified siRNA demonstrated an improved safety profile up to 10 mg/kg dose^74^. Base modification is not widely used for siRNA modification yet, these modifications may offer prospective advantages and opportunities to enhance therapeutic potential. Commonly used base analogs include 5-methylcytidine, N6-methyladenosine, and various uridine and cytidine residues^75^]. The most utilized nucleoside modification in siRNA is the incorporation of a 5′-triphosphate cap, which has been shown to enhance siRNA activity by facilitating its loading onto the RNA-induced silencing complex (RISC)^76^. Another nucleoside modification involves attaching a cholesterol moiety to siRNA, improving its cellular uptake, and promoting its accumulation in target tissues^77^].

### 2.10 Therapeutic delivery of siRNA

The development of delivery strategies has been a major bottleneck on the way toward using siRNA in in-vivo and clinical applications. Clinical approval of siRNA therapeutics has been largely limited to siRNA conjugates with GalNAc for targeted hepatocyte delivery^78^]. Another major approach for siRNA beyond the liver is lipid nanoparticle (LNP) which protects siRNA from enzymatic degradation and enhances cellular uptake. A notable example is FDA-approved siRNA drug patisiran (ONPATTRO™), which is used to treat heredity transthyretin-mediated amyloidosis (hATTR)^79^. However, the large LNP size can hinder tissue penetration and lead to the potential accumulation in the RES which raises concern about biodistribution and immunogenicity. As of March 2023, the FDA approved 15 oligonucleotide therapeutics including RNAi therapies^80^]. Most approved RNAi therapeutics are limited to liver disease due to the effective delivery methods like GalNAc conjugation and lipid nanoparticles (LNPs) that facilitate precise delivery to hepatocytes. The most remarkable finding in delivery technology since the discovery of RNAi was the replacement of LNP-based delivery by GalNAc conjugation as the predominant method in 2017. However recent advancement has expanded the scope of RNAi treatment beyond the live disease. For example, Nedosiran for PH1, a kidney-related disorder, demonstrates the versatility of RNAi technologies in addressing diseases beyond the liver^81^. Functionalized nanoconjugate comprising survivin siRNA encapsulated with biodegradable PLGA nanoparticles, which were PEGylated and further decorated with GalNAc ligands have been engineered to target HCC cells. Thess GalNAc@PEG@*siRNA*-PLGA nanoconjugate demonstrated specific binding to ASGPRs on HCC cells which further significantly reduced the expression of survivin protein which is essential for tumor cell survival^82^. In the same direction, the thermosensitive hydrogel is utilized to achieve sustained release of GalNAC-siRNA/DP7-C nanoparticles. This nano formulation effectively regulates Pin1 expression in HCC cells and inhibited tumor suppression and showed its therapeutic potential in HCC^83^]. Selenium nanoparticles (SeNPs) are particularly promising anticancer drug carriers as their core material offers interesting synergistic effects on cancer cells^84^]. Layer by layer assembled Se nanocomplex using electrostatic interactions between siRNA and chitosan on a SeNP core^84^[. These nanoparticles significantly induce apoptosis in H1299 lung carcinoma cells, promising siRNA carriers in cancer therapy. Hung et al. developed an azoreductase-activated nanocomplex encapsulating DOX and HIF-1alpha siRNA. The primary ligand, 4,4′-azobisbenzoic acid of this nanocomplex is reduced by the azoreductase hypoxic tumor microenvironment. In hypoxic tumors, DOX and siRNA are released, this targeted delivery effectively reduces HIF-1 alpha expression^85^. MDR often leads to chemotherapy failure, primarily due to the overexpression of P-gp, a drug efflux pump. A hyaluronic acid coated, pH/redox dual responsive nanosystem has been developed for co-delivery of DOX and GCNS siRNA. This targeted delivery system enhanced drug accumulation in CD44 overexpressing cancer cells. It downregulated the expression of P-gp expression by silencing GCN5 and significantly inhibits tumor growth while reducing systemic toxicity^86^]. Fluorescent nanodiamonds are nanoscale diamond articles with an optical property. FNDs can deliver siRNA in a xenograft model. Image analysis indicated minimal accumulation of intravenously administered FNDs in the tumor, suggesting that direct intertumoral delivery may enhance efficacy. This method resulted in a 28-fold suppression of the oncogene associated with Ewing tumors, indicating a potential for drug delivery^87^]. Mesoporous silica nanoparticles (MSNPs) are a potential carrier in cancer research. A novel zwitterionic (2-methacryloyloxyethyl phosphorylcholine (MPC) PEI coated core–shell (Fe3O4@SiO2 MSNPs) was designed to minimize unspecific protein adhesion. This system effectively delivered daunorubicin and Twist siRNA and induced cytotoxicity in Ovcar8 of up to 50 %. These engineered low-fouling nanoparticles exhibit controlled drug release with promising therapeutic potential^88^]. Apart from NP mediated delivery system there are other alternate methods like conjugation of siRNA to ligands or embedding macromolecules with biodegradable matrix. Such a method provides better sustained release and higher precision^89^]. Ligand-linked siRNA delivery, known as antibody–siRNA conjugates (ARC), is a promising platform like Antibody Drug Conjugates (ADC). Cuellar et al. developed an ARC using THIOMAB technology, but its efficacy was limited to high-antigen-expressing systems due to poor cytoplasmic translocation. Protamine-based linkers improved translocation but posed aggregation risks^90^].

### 2.11 Optimizing siRNA design

Bioinformatics tools are essential for mitigating siRNA off-target effects by analyzing and predicting potential unintended interactions. To reduce off-target effect guide and passenger strands should be checked against the Ref-Seq RNA database using BLAST(https://www.ncbi.nlm.nih.gov/refseq/)^91^. It helps to find that alignments are not due to the chance. Off-target effects are considered tolerable if more than 15 nucleotides out of 19 are matched with designed siRNA^91^]. Another criterion is if query coverage with other genes is < 78 %. siRNA designing tool siDirect (https://sidirect2.rnai.jp/) applies mismatch tolerance parameter to evaluate off-target effect with ≥ 3 mismatches in sense and antisense strand strands for the high specificity with the target site^92^). Thermodynamic properties significantly influence off-target effects. siDirect applies a melting temperature (Tm) of < 21.5 °C for the seed region duplex, further reducing off-target interactions. Algorithms like BLAST and RNAhybrid (https://bibiserv.cebitec.uni-bielefeld.de/rnahybrid), coupled with transcriptome-wide analysis, are valuable for identifying off-target sites^93^. However, these algorithms have limitations including prolonged processing times and a frequent exclusion of crucial biological factors. Consequently, they may fail to detect biologically significant miRNA-like off-target effects, particularly those arising from the partial Watson-Crick complementarity within the short seed sequence. To address this challenge many siRNA design tools now incorporate miRNA-like off-target prediction and partial complementarity filtering. Additionally, standalone tools such as Genome-wide Enrichment of Seed Sequence matches (GESS) https://fgr.hms.harvard.edu/online-gess and RIsearch2 (https://rth.dk/resources/risearch/) are also available[94], [95]. Furthermore, miRNA-target prediction algorithms can be integrated into workflows to further enhance miRNA-like off-target prediction, enabling the design of more precise and effective siRNA therapeutics. The secondary structure of mRNA is another determinant of siRNA efficacy. Native mRNAs have a secondary structure that can be detected by software like Mfold (https://www.unafold.org/mfold/applications/rna-folding-form.php). Gredell et al. found that siRNAs are more effective on target sites with four consecutive unpaired bases at 5′to 3′ ends^96^]. RNAxs (http://rna.tbi.univie.ac.at/cgi-bin/RNAxs/RNAxs.cgi) developed by Tafer et al. designs siRNAs based on target accessibility^97^].

### 2.12 Tools for siRNA designing

The design of effective siRNAs was first outlined by Elbashir et al. in 2001. It involves creating siRNAs that are 19–29 nucleotides in length, with a preference for shorter siRNAs (19–25 nucleotides) to avoid nonspecific binding and immune responses. siRNAs should have a GC content between 30–53 %, with specific attention to nucleotide composition for optimal gene silencing^98^. To evade immune activation, chemically modified siRNAs (mentioned in the above section) or those with fewer than 24 base pairs are preferred. Key design considerations include avoiding secondary structures, maintaining proper duplex formation, and ensuring strand bias by favoring A/U at the 5′-end of the antisense strand. Effective siRNAs target regions within the open reading frame, preferably 50–100 nucleotides downstream of the start codon, avoiding untranslated regions or areas close to the start codon. The selection of target sites is critical, especially in applications like oncology and antiviral therapies, where targeting conserved and functionally essential regions is crucial^99^].

**Fig. 7:**
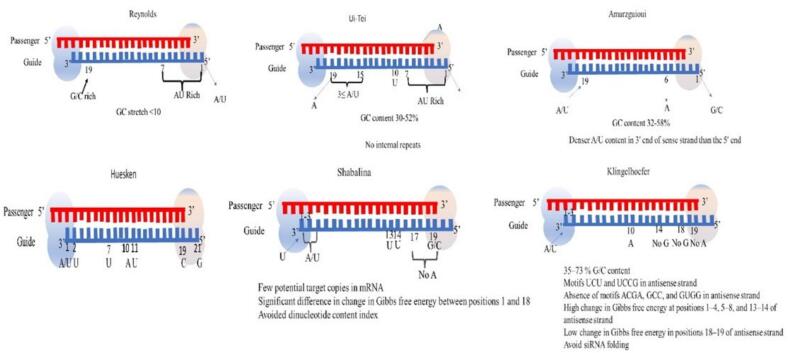
Effective siRNA design features based on sequence requirements and suggested modified bases for desired gene silencing.

Designing highly effective siRNA is an important parameter. All existing web portals just consider the aforementioned parameters, while ignoring other crucial variables that must be considered in order to create siRNAs with enhanced efficiency Fig. 7. The critical aspects to consider are the binding effectiveness and stability of the proposed siRNAs in a cellular condition. Therefore, it is crucial to investigate the stability of the designed siRNA targeting the desired gene. To achieve this objective, the web portal that is now freely available can be used to perform chemical modification and assess the stability of each siRNA that has been modified chemically. Ensuring the stability of siRNA is essential for effectively reaching the target site. Prior to doing in vivo or in vitro research, it is necessary to perform in silico binding analysis[,[34], [100], [101]].

The knockdown efficiency of siRNAs is found to be strongly influenced by their sequences. Researchers supported the experimentally derived guideline that defined the attributes of highly efficient siRNAs^102^. This guideline is known as the Ui-Tei rule. The siRNA chosen according to the Ui-Tei rule must meet the following four conditions at the same time: (1) Have either an A or U at position 1 from the 5′ terminus of the siRNA guide strand, (2) Have a G or C at position 19, (3) Have an AU richness (AU ≥ 4) in positions 1–7, and (4) Not have a long GC stretch of ≥ 10. With the exception of (4), the Rule is that the functional siRNA exhibits unequal stability in the 5′ and 3′ terminals. Additional research groups have also provided evidence supporting the principles of highly efficient siRNAs, known as the Reynolds rule and the Amarzguioui rule^103^. The melting temperature (Tm), a thermodynamic parameter that measures the stability of RNA duplex formation, exhibited a significant positive connection with the occurrence of seed-dependent off-target effects. In parallel machine learning has emerged as a powerful tool in optimizing siRNA designing. The most recent advance is training artificial neural networks (ANNs) on extensive datasets of chemically modified siRNAs to predict their silencing efficiency based on sequence and modification patterns. This data-driven approach helps in the identification of potent siRNA with tailored chemical modification, streaming the development of effective siRNA therapeutics.

There are lots of online tools for siRNA designing. Some of them are updated regularly whereas others are not supported anymore. Several publicly available software tools have been developed for selecting functional siRNAs, including the Ui-Tei rule^102^, Reynolds rule^104^, Amarzguioui rule^103^, Tuschl rule^98^, and various combinations thereof. These tools are commonly and extensively utilized. To exclude the non-target genes that had a very close match, a homology search was conducted using the BLAST search algorithm in many software programs. However, due to the limited accuracy of BLAST search for small sequences such as siRNAs, other very precise homology search engines like siDirect, WU-BLAST, and Bowtie are commonly employed. Out of all the options, siDirect 2.0 is likely to provide the most precise results. In addition, certain software programs consider supplementary characteristics, such as the secondary structure of mRNA, alternative splicing, or the specific sequence pattern that may trigger an immune response to an RNA virus. siDirect 2.0 does not take these features into consideration. In this part, we introduce some of the best online software and their characteristics. Their advantages and limitations are shown in Table 1.

**Table 1:** Various online available tools for the designing of siRNA with their website details, advantages, and disadvantage.

**Software Tool**	**Website**	**Advantages**	**Disadvantages**
siDirect	https://sidirect2.rnai.jp/	Integrates multiple criteria for effective siRNA design, such as thermodynamic properties and target site accessibility to design siRNA which specifically target mRNA.	Limited customization options for advanced users
T RNAi Designer	https://rnaidesigner.thermofisher.com/	User-friendly interface and straightforward workflow Provides comprehensive guidelines and support for siRNA design	Limited control over some advanced design parameters
DSIR (Designer of siRNA)	https://biodev.cea.fr/DSIR/reference.html	Powerful algorithm based on empirical data to predict effective siRNAs Offers batch processing for high-throughput siRNA design	The interface may be less user-friendly for those not familiar with siRNA design
siRNA Target Finder	https://www.ambion.com/ l	Developed by a leading provider of life science tools Comprehensive database and integration with other RNAi products	Some advanced features might be behind a paywall or require institutional access
RNAxs	http://rna.tbi.univie.ac.at/	Based on mRNA target site accessibility Incorporates multiple predictive models for effective siRNA design	The interface can be complex for new users Requires familiarity with RNA secondary structure prediction
Dharmacon siDESIGN Center	https://horizondiscovery.com	Comprehensive siRNA design tool with detailed instructions High-quality siRNA libraries available for purchase	Limited free features compared to premium options
Clontech RNAi Designer	https://www.hsls.pitt.edu/obrc	Integrated with Clontech's RNAi products Provides detailed design parameters	Less flexibility in design compared to some other tools
siRNA Design (IDT)	https://www.idtdna.com/site/order/designtool/index/DSIRNA_CUSTOM)	User friendly results and explanations Covering different patterns of bases and asymmetrical end stability Including a new form of siRNA design with sufficient guide and explanations	The interactive tutorial is not updated(b) Only designing 25-nt siRNAs in custom approaches
RNA wizard	https://www.invivogen.com/sirnawizard/	Designing siRNAs in various sizes shRNA design and offering efficient and novel vectorsPerforming two kinds of blast: (1) blast against mRNAdatabase and (2) blast against the miRNA database Considering optimal internal stability of siRNAs Excluding immunogenic sequences	Only the sequence of the query is accepted
siMAX siRNA Design	https://eurofinsgenomics.eu/en/dna-rna-oligonucleotides/oligo-tools/sirna-design-tool/	Targets specific genes, reducing the likelihood of off-target effects. Offers a wide range of siRNAs for various genes, providing comprehensive options for researchers.	Pre-designed siRNAs may not be available for all genes or specific isoforms.
Gene Link siRNA Design	https://www.genelink.com/sirna/shRNAi.asp	Provides online tools like RNAi Explorer™ and Oligo Explorer™ for predictive design based on existing literature. Allows customization based on specific sequences and experimental needs. The Guaranteed RNAi Explorer™ Kit ensures performance under specific conditions.	Relies on predictive algorithms, which may not always yield optimal results. Custom siRNA design and synthesis can be expensive.

### 2.13 Therapeutic application of siRNA in cancer

The development of siRNA-based anticancer nanotherapeutics has progressed significantly through various phases of clinical trials. Several studies have explored the potential of siRNA in targeting specific oncogenes, demonstrating promising preclinical results but facing challenges in clinical translation[105], [106]], as shown in Table 2.

**Table 2:** Detailed Summary of siRNA therapeutics targeting oncogenes and tumor Suppressor Genes in Cancer: target gene, specific cancer, observations, clinical trial phases, identifier, and Sponsorship.

**siRNA**	**Target gene**	**Cancer**	**Observation**	**Clinical Trail status**	**Identifier**	**Sponsor**
NUDT21 SiRNA	NUDT 21	**Retinoblastoma (RB)**	The primary outcome measure is Incidence of Treatment-Emergent Adverse Events	Early Phase 1	NCT06424301	Eye & ENT Hospital of Fudan University^107^]
STP705	TGF-β1 and COX-2	**Solid tumor and Cutaneous squamous cell carcinoma (in situ) skin cancer (isSCC).**	Phase I included 25 patients with cutaneous squamous cell carcinoma across five cohort doses (10,20,30,60, and 120 μg), and 76 % of participants achieved complete histological clearance. Interim data from 2022 showed that 78 % of patients achieved clearance. In 2023 after discussing with the FDA, Sirnaomics decided to start a phase III study.	Phase I/ Phase II	NCT04844983 NCT04293679	Sirnaomics[,[108], [110]^111^]
STP707	TGF-β1 and COX-2	**Solid tumor**	In phase one study it showed a good safety profile and limited low-dose toxicity. It reduces significantly tumor size up to 70 %.	Phase I	NCT05037149	Sirnaomics^109^]
TKM‐080301	PLK1	**Hepatocellular Carcinoma**	Preliminary antitumor activity showed 46.2 % of patients achieved stable disease, while 23.1 % had a partial response. The median overall survival was 7.5 months, suggesting limited efficacy as a single-agent	Early-phase study	NCT01262235	Arbutus Biopharma Corporation^121^]
APN401	Cbl-b	**Pancreatic cancer and Colorectal cancer**	APN401 infusions were well tolerated. One patient in the first, three in the second, and two in the third cohort developed grade 2 chills at the completion of the infusion. These responded to meperidine. Grade 3 or 4 toxicities were not observed.	Phase I/ Phase II	NCT03087591	Wake Forest University Health Sciences^128^]
NBF-006	GSTP	**Non-Small Cell Lung Cancer, Colorectal cancer, Pancreatic cancer**	The drug was well tolerated, with no grade 4–5 adverse events or dose-limiting toxicities. The most common side effects were grade 1–2 arthralgia, diarrhea, fatigue, and vomiting.	Phase I	NCT03819387	Nitto BioPharma, Inc.^129^]
siG12D-LODER	KRAS G12D	**Pancreas cancer**	Upon intratumoral injection, siG12D is released locally, thereby preventing the translation of KRAS proteins and potentially inhibiting the growth of tumor cells overexpressing KRAS. KRAS, a member of the small GTPase superfamily, is mutated in over 90 % of human pancreatic ductal adenocarcinomas (PDAC) and is associated with tumor cell proliferation and reduced survival.	Phase 2	NCT01676259	Silenseed Ltd^7^]
Atu027	PKN3	**Pancreatic adenocarcinoma**	Upon administration, catiogenic and fusiogenic lipids promote anti-PKN3 siRNA Atu02 uptake by tumor cells; the siRNAs moieties are subsequently released once inside the cell. The siRNAs bind to PKN3 mRNAs, which may result in the inhibition of translation and expression of the PKN3 protein and, so, growth inhibition of tumor cells that overexpress PKN3.	Phase I/ Phase II	NCT01808638	Silence Therapeutics GmbH^130^]
CALAA-01	RRM2	**Refractory cancer**	Determine the safety, toxicity, and the maximum tolerated dose (MTD) of CALAA-01 when administered intravenously to patients	Phase 1	NCT00689065	Calando Pharmaceuticals^131^]
EphA2 siRNA-DOPC	EphA2	**Prostate cancer**	Preclinical studies demonstrated good tolerance and potential efficacy in reducing tumor growth. The trial seeks to evaluate the safety and preliminary efficacy of this promising therapeutic strategy in human subjects.	Phase 1	NCT01591356	M.D. Anderson Cancer Center^132^]
DCR-MYC	MYC.	**Solid tumors, Multiple myeloma, or lymphoma**	DCR-MYC demonstrated an acceptable safety profile and evidence of target engagement in these early-phase trials, but further development is needed to determine its clinical efficacy in advanced cancers driven by MYC dysregulation. The trials were terminated early by the sponsor for undisclosed reasons	Phase I	NCT02110563	Dicerna Pharmaceuticals, Inc., a Novo Nordisk company^106^]
ARO-HIF2	HIF2α)	**Renal Cell Carcinoma**	ARO-HIF2 was generally well-tolerated, with the most common treatment-emergent adverse events (TEAEs) being fatigue (50 %), dizziness (26.9 %), dyspnea (23.1 %), and nausea (23.1 %).	Phage I	NCT04169711	Arrowhead Pharmaceu ticals[113], [114]]

GSTP: Glutathione S-transferase Pi; KRAS G12D: Kirsten Rat Sarcoma viral oncogene homolog (G12D mutation); PKN3: Protein Kinase N3; RRM2: Ribonucleotide Reductase M2; EphA2: Ephrin type-A receptor 2; MYC: MYC proto-oncogene (c-Myc); AR: Androgen Receptor; HIF2α: Hypoxia-inducible factor 2-alpha.

**NUDT21 SiRNA** was developed by the Eye & ENT Hospital of Fudan University. NUDT21 siRNA therapy works by promoting tumor apoptosis through the regulation of the 3′UTR plus tail of the SMC1A gene. SMC1A holds sister chromatids during the cell division, reducing the proliferative activity in tumor cells. This study started in Dec 2024 and explores the effectiveness of NUTD21 siRNA in retinoblastoma patients. The dosing regimen involves intravitreal injections of the targeted drug at a dosage range of 100–1000 μg, administered on day 1 of week 1 and week 3. Clinical assessments are conducted weekly, with continuous follow-up until 12 weeks post-treatment. In addition to monitoring treatment response, the study evaluates drug presence in blood samples and aqueous humor, immune response, and potential adverse effects^107^].

**STP707** developed by Sirnaomics is nanoparticle-formulated siRNA therapeutics. It targets TGF-β1 and COX-2, a key regulator in tumorigenesis and inflammation. Preclinical models demonstrated robust gene knockdown in multiple organs (liver, lung, and xerograph models). In phase I clinical trials were conducted in patients with solid tumors. STP707 showed a good safety profile with low dose-limiting toxicities. 74 % of evaluable patients demonstrated the best response of stable disease and several patients exhibited a reduction in tumor burden. In a preclinical study, intravenous administration of STP707 reduced the expression of TGF-β1 and COX-2, resulting in anti-tumor activity. Additionally, combination therapy studies in mouse liver orthotopic tumor models revealed that STP707 in combination with immune checkpoint inhibitors led to a 40–60 % reduction in tumor size[108], [109]].

**STP705** is an siRNA therapeutic drug targeting TGF-β1 and COX-2. The aim of STP705 is to reduce tumor growth and enhance immune response. Phase I included 25 patients with cutaneous squamous cell carcinoma across five cohort doses (10,20,30,60, and 120 μg), and 76 % of participants achieved complete histological clearance. Interim data from 2022 showed that 78 % of patients achieved clearance. In 2023 after discussing with the FDA, Sirnaomics decided to start a phase III study. This trial includes a single-dosage study focusing on a specific subgroup of patients as part of a larger, prospective study[110], [111]].

**NCT05902520** Phase 1 clinical trial (NCT05902520), sponsored by AgonOx, Inc. The therapy primarily involves adoptive cell therapy (ACT) using tumor-infiltrating lymphocytes (TILs). However, one experimental arm includes siRNA-mediated PD-1 knockdown, which enhances TIL function by reducing immune suppression. The main objective of this trial is safety assessment through adverse events along with tumor response. A total of 18 patients with advanced solid tumors included underwent lymphodepleting chemotherapy before TIL and IL-2 infusion. This trial is currently open to enroll participants^112^].

**ARO-HIF2** in phase 1B clinical trial, designed to target hypoxia-inducible factor-2α (HIF2α). This trial included 24 patients for the treatment of clear cell renal cell carcinoma. The trial was sponsored by Arrowhead Pharmaceuticals. HIF-2α contributes to tumor cell proliferation, survival, and immune evasion for therapeutic intervention. The trial focuses on determining a safe and effective dose. The most common adverse effects were fatigue, dizziness, dyspnea, and nausea suggesting a manageable safety profile. The study is further recommended for the Phase II trial[113], [114]].

**Atu027**, a siRNA-based lipid nanoparticle designed to knock down protein kinase N3 (PKN3), is linked to angiogenesis and tumor metastasis. PKN3 physically interacts with Rho-family GTPases, and preferentially with RhoC, a known mediator of tumor invasion and metastasis. The knockdown of PKN3 in orthotopic mice demonstrated reduced tumor size and impaired tumor metastasis Atu027^115^, was tested in a Phase I trial involving 34 patients with advanced solid tumors. Atu027 was administered intravenously and showed well tolerated by patients with over 90 % adverse effect being grade 1 and grade 2 without dose-limiting toxicities up to the maximum tested dose of 0.336 mg/kg, and some patients experienced disease stabilization or regression of pulmonary metastasis^116^]. ATU027 response was substantially more significant in patients with PC^117^. Therefore, a Phase II clinical trial was conducted for ATU027 combined with gemcitabine (GEM) in patients with advanced pancreatic carcinoma. The aim of the study was to evaluate the efficacy and pK of combination therapy. Change in VEGFER-1 was also observed indicating a potential biomarker for the assessment of ATU027 efficacy^118^].

**ALN-VSP02**, developed by Alnylam Pharmaceuticals, contains siRNAs targeting kinesin spindle protein (KSP) and VEGF. This gene is responsible for cell division and new blood vessel formation in tumors. By inhibiting the expression of KSP and VEGF tumor cell progression can be halted. The Phase I trial involved a dose-escalation phase with 41 patients with advanced solid tumors (liver) enrolled between March 2009 and August 2011. Patients received an intravenous infusion of ALN VSP02 at a dose range from 0.1 to 1.5 mg/kg, followed by an expansion phase. The trial reported no significant safety concerns, and some patients experienced prolonged tumor stabilization. Molecular analysis of the sample confirmed the presence of residual siRNA and mRNA at the target site. A patient with endometrial cancer received a complete response with full tumor regression after 50 doses over 26 months. Three patients exhibit stable tumors after 17 to 36 doses over 8 to 18 months However, due to the small sample size and tumor heterogeneity, no definitive correlations between molecular suppression and clinical response were established^119^].

**TKM-PLK1** developed by Tekmira Pharmaceutics, is a siRNA-based therapeutic targeting POLO-like kinase 1. PLK1 is a serine/threonine kinase that plays an essential role in the cell cycle(mitosis). Overexpression of PLK1 led to the uncontrolled cell death and tumorigenesis^120^. Initial Phase I trial assessed its safety and efficacy in patients with gastrointestinal neuroendocrine tumor and adrenocortical carcinoma demonstrated favorable toxicity with moderate anti-tumor activity. Encouraged by this result Phase I/II clinical trial start by Tekmira Pharmaceutics in 2014 to evaluate TKP-PLK 1 in advanced hepatocellular carcinoma. TKM-PLK1 was well tolerated but antitumor activated was limited it did not show an improved survival rate compared to the control. Consequently, future evaluation of TKM-PLK 1 a single therapy agent in larger randomized trials was not pursued^121^].

**siG12D LODER**, the first siRNA anticancer nano therapy administered intra-tumorally, targets the G12D-mutated KRAS gene, implicated in pancreatic ductal adenocarcinoma. The Phase I trial demonstrated a favorable safety profile with no dose-limiting toxicities, and superior clinical responses were observed when combined with standard chemotherapy. Building upon these promising results, a phase II study was initiated in 2017 to further evaluate the efficacy of siG12D LODER in combination therapy in patients with locally advanced pancreatic cancer. This open-label, randomized control trial includes 80 participants who received either repeated doses of 2.8 mg siG12D LODER every 12 weeks in combination with chemotherapy (paclitaxel, gemcitabine, etc.) or chemotherapy alone. The primary outcome measure was the objective response rate Overall, these trials highlight the potential of siRNA-based therapies in cancer treatment, though challenges such as small sample sizes and the need for further studies remain. These therapies offer a promising avenue for targeting specific genetic pathways involved in cancer progression. As of the latest updates, this Phase II trial is ongoing, with recruitment status listed as active but not recruiting^7^].

**NBF-006** is a lyophilized lipid nanoparticle (LNP) encapsulating siRNA that targets glutathione-S-transferase P (GSTP), a key regulator of the RAS signaling pathway proteins. GSTP is notably overexpressed in various cancers, particularly those with KRAS mutations, such as lung, colorectal, and pancreatic cancers. In preclinical studies using a KRAS mutant non-small-cell lung cancer (NSCLC) xenograft model, NBF-006 demonstrated significant tumor suppression and prolonged survival, with 70–80 % of the administered dose being internalized by tumor tissues and was well tolerated by the animal models. Following these promising results, NBF-006 entered a Phase I clinical trial to assess its impact on patients with NSCLC, colorectal, or pancreatic cancer, with or without KRAS mutations. The treatment involved intravenous administration once weekly for four weeks, repeated every six weeks, with longer treatment durations for patients with KRAS mutant cancers. As of the latest updates study has been completed with the enrollment of 49 patients and the results have not been published publicly^122^].

**EphA2-siRNA-DOPC**, known as EPHARNA, is another recent siRNA-mediated nanotherapeutic targeting the EphA2 receptor. It is a tyrosine kinase protein receptor upregulated in several cancers including breast, ovary, skin, colon, and brain. This increased expression is associated with enhanced tumorigenicity, evasion of metastasis, and apoptosis^123^. In clinical trials EPHARNA, delivered via liposomal nanoparticles, has shown anti-angiogenic effects and significant tumor growth reduction in both in vitro and in vivo studies. When combined with paclitaxel, it further inhibits tumor growth. Toxicological studies indicated no adverse events at doses of 75–225 mcg/kg, and the therapy entered a Phase I trial in 2015 for patients with advanced metastatic solid cancers^124^].

**CALAA-01** targeted the M2 ribonucleotide reductase subunit (RRM2) and showed potential in reducing tumor growth in preclinical models, but the clinical trial faced challenges with tolerability. RRM2 is an essential enzyme for DNA synthesis and repair by producing dNTP. Overexpression of RRM2 can produce cancer metastasis by activating the PI3K/AKT signaling pathway to induce cell invasion, migration, and angiogenesis^125^]. The trial for CALAA-01 was conducted on 15 patients suffering from different types of cancer. The dose was administered intravenously, and it ranged from 3-30 mg/m^2^. Early results were very promising, showing a significant reduction in tumor size. However, dose-limiting toxicity emerged at 30 mg/m^2^, with two patients experiencing severe adverse effects even after reducing the dose to 24 mg/m^2^, this led to the discontinuation of the trial. Despite these challenges, this clinical trial provides valuable insight into the pharmacokinetics and biodistribution of siRNA which highlights the requirement of an improved delivery system for the systemic delivery of therapeutic siRNA development^126^].

**DCR-MYC**, a Dicer substrate siRNA (DsiRNA) developed by Dicerna Pharmaceuticals, targets c-Myc overexpressed in cancerous cells. C-Myc is a transcription factor, that regulates genes involved in cell proliferation, survival, and metabolism. It aberrantly expressed in over 70 % human cancer^127^]. DCR-MYC was tested in a Phase I trial starting in 2014, involving patients with solid tumors, multiple myeloma, Non-Hodgkin’s Lymphoma, and pancreatic neuroendocrine tumors. Despite showing a favorable safety profile, the trial was terminated due to unmet clinical expectations^106^]. A dose-escalation study of DCR-MYC in 19 patients with advanced tumors showed dose-proportional PK changes and common treatment-related adverse events like fatigue and nausea. Tumor shrinkage and metabolic responses were observed in a number of patients. The pre-clinical results did not meet the expectations of researchers. Paired tumor biopsies showed degree of MYC gene expression was below the threshold level. These findings indicate that either the level of MYC knockdown was inadequate, or tumors developed compensatory resistance mechanisms, warranting further investigation^106^].

## 3 Limitations of the therapeutic use of siRNAs

Several research groups have shown the therapeutic potential of siRNA in cancer treatment, yet efficient cellular uptake and stable delivery of siRNAs to their target genes is a major hurdle. These hurdles must be considered so that siRNA technology can revolutionize cancer therapy. Intracellular delivery to specific target genes is also associated with the clinical use of siRNAs in humans. Besides, naked synthetic siRNAs are short-lived and have the risk of being degraded by nuclease enzymes and rapidly being excreted out of the body. Despite these hurdles, systematically delivered siRNAs may induce an innate immune response against them. Sometimes, endosomal retention of delivered siRNAs may induce TLR7/8-mediated immune responses. In this context, siRNA technology cannot be used significantly against cancer without overcoming these issues. Recent clinical trials showed the promising potential of siRNA against cancer therapy. However, there is a requirement to explore more potent biomarkers of cancer to develop siRNA against them.

## CRediT authorship contribution statement

**Jyoti Singh:** Writing – original draft, Software, Methodology. **Abdulaziz S. Saeedan:** Writing – original draft, Software, Resources, Conceptualization. **Gaurav Kaithwas:** Writing – review & editing, Supervision, Resources, Project administration, Methodology, Funding acquisition, Conceptualization. **Mohd Nazam Ansari:** Writing – review & editing, Supervision, Resources, Project administration, Funding acquisition, Conceptualization.

## Funding

“The authors extend their appreciation to Prince Sattam bin Abdulaziz University for funding this research work through the project number (PSAU/2024/03/31724)”.

## Declaration of Competing Interest

The authors declare that they have no known competing financial interests or personal relationships that could have appeared to influence the work reported in this paper.
